# Atypical centromeres in plants—what they *can* tell us

**DOI:** 10.3389/fpls.2015.00913

**Published:** 2015-10-26

**Authors:** Maria Cuacos, F. Chris H. Franklin, Stefan Heckmann

**Affiliations:** School of Biosciences, University of Birmingham, Birmingham, UK

**Keywords:** centromere, kinetochore, holocentric chromosomes, neocentromeres, cenH3, plants, mitosis, meiosis

## Abstract

The centromere, visible as the primary constriction of condensed metaphase chromosomes, is a defined chromosomal locus essential for genome stability. It mediates transient assembly of a multi-protein complex, the kinetochore, which enables interaction with spindle fibers and thus faithful segregation of the genetic information during nuclear divisions. Centromeric DNA varies in extent and sequence composition among organisms, but a common feature of almost all active eukaryotic centromeres is the presence of the centromeric histone H3 variant cenH3 (a.k.a. CENP-A). These typical centromere features apply to most studied species. However, a number of species display “atypical” centromeres, such as holocentromeres (centromere extension along almost the entire chromatid length) or neocentromeres (ectopic centromere activity). In this review, we provide an overview of different atypical centromere types found in plants including holocentromeres, *de novo* formed centromeres and terminal neocentromeres as well as di-, tri- and metapolycentromeres (more than one centromere per chromosomes). We discuss their specific and common features and compare them to centromere types found in other eukaryotic species. We also highlight new insights into centromere biology gained in plants with atypical centromeres such as distinct mechanisms to define a holocentromere, specific adaptations in species with holocentromeres during meiosis or various scenarios leading to neocentromere formation.

## Centromere Types

Centromeres are chromosomal loci where kinetochores assemble. Kinetochore proteins mediate cell cycle regulation, sister chromatid cohesion, spindle microtubule attachment and chromosome movements (Lermontova et al., 2014). These functions are essential for genome stability by mediating faithful mitotic and meiotic chromosome segregation. Any failure leads to chromosome missegregation and ultimately genome instability.

Kinetochore establishment and centromere maintenance in active eukaryotic centromeres generally depends on the presence of the centromeric histone H3 variant cenH3 (also called CENP-A in mammals; De Rop et al., 2012). Although essential for genome integrity, contrary to expectation centromeric cenH3 localization is not specified by centromere specific DNA sequence(s) except in budding yeast (Clarke and Carbon, 1985). It is rather determined epigenetically. Centromere loci and centromeric DNAs are highly diverse varying dramatically in size and sequence composition between species. Centromeres can range in size from the 125 bp “point” centromeres in budding yeast up to mega bp-sized “regional” centromeres in humans and plants. In the most extreme case, poly- or holocentromeres can even extend over the entire chromosome length.

Although centromeric DNAs are not conserved often plant centromeres contain distinct satellite DNA sequences and families of long terminal repeat (LTR) retrotransposons (Houben and Schubert, 2003; Neumann et al., 2011). However, these repeats are neither necessary nor sufficient for centromere activity since gain of new centromeric activity over unique DNA sequences can occur (Nasuda et al., 2005; Han et al., 2006).

Centromeric DNAs are one of the fastest evolving sequences in eukaryotic genomes (Bensasson, 2011; Melters et al., 2013). It is interesting that such an essential and functionally conserved chromosomal locus has so rapidly evolved with regards to its structure, extension and DNA sequence composition. For instance, (i) the sequence composition and centromere extension vary dramatically between closely-related species, e.g., *Solanum* (Zhang et al., 2014) or *Oryza* species (Yi et al., 2013), and even between centromeres within one species, e.g., *Pisum sativum* (Macas et al., 2007), (ii) different centromere types, e.g., mono- and holocentromeres, have evolved between different insect lineages (Drinnenberg et al., 2014) and even between closely-related dodder species (Pazy and Plitmann, 1994, 1995), or (iii) albeit functionally similar, unconventional centromeres, e.g., cenH3- and CENP-C-independent insect holocentromeres (Drinnenberg et al., 2014) or “meta-polycentric” centromeres in *Pisum* or *Lathyrus* (Neumann et al., 2012, 2015), and unconventional kinetochores, e.g., kinetoplastid kinetochores devoid of any conventional components (Akiyoshi and Gull, 2014), have evolved.

Studies on “atypical” plant centromeres such as neocentromeres or holocentromeres have contributed to our general knowledge of the structure, regulation and function of centromeres. In this review, we focus on such unusual centromere types in plants, highlight recent discoveries and discuss their implications.

## Holocentromeres

**FIGURE 1:**
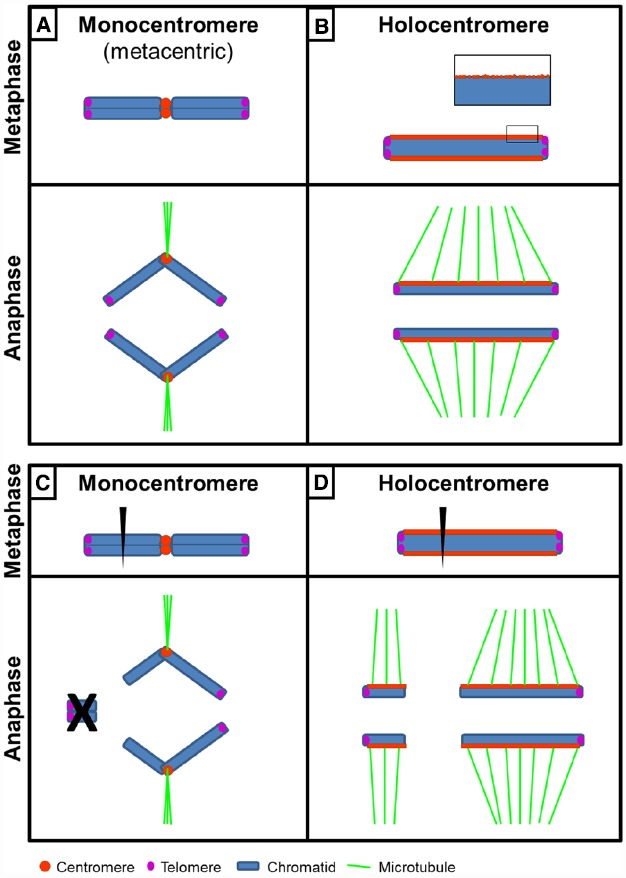
**Structure and behavior of a monocentric and a holocentric chromosome. (A)** A metacentric chromosome shows a primary constriction during metaphase. During anaphase chromatids move as V-shaped structures due to microtubule attachment to the size-restricted centromere. **(B)** A holocentric chromosome shows an almost chromosome-wide centromere extension and no primary constriction during metaphase. Sister chromatids are not discernible. During anaphase spindle microtubule attachment to the holocentromere results in chromatids moving as linear bars parallel to the spindle. Inset, various centromeric subdomains fuse to one functional composite linear holocentromere at metaphase. **(C)** Breakage of a monocentric chromosome results in loss of the acentric chromosome fragment during anaphase, whereas **(D)** after chromosome breakage of a holocentric chromosome both fragments retain kinetic activity due to the almost chromosome-wide centromere extension and thus can be transmitted. Note absence of telomeric repeats at broken chromosome ends. In case of holocentric chromosomes of *Luzula elegans*, rapid telomere-mediated “chromosome healing” occurs (Jankowska et al., 2015).

Most studied organisms possess one size-restricted centromere (monocentromere) per chromosome (Figure 1A). However, in various species so-called holocentromeres (“holo-” from Greek: entire) initially described by Schrader (1935), characterized by an almost chromosome-wide extension occur (Figure 1B). They are also called diffuse centromeres or polycentromeres—for the rest of the review we will use the terms holocentromere or holocentric chromosome. Holocentromeres evolved by convergent evolution in diverse eukaryotic lineages including green algae, invertebrates, and plants (Melters et al., 2012). Around 800 species as diverse as nematodes, spiders, and sedges are reported to possess holocentromeres.

## Chromosome Classification: Monocentromere vs. Holocentromere

If a given chromosome is comparatively large, classical cytogenetic techniques are applicable for the classification as holo- or monocentric. In the case of a holocentromere, a mitotic metaphase chromosome lacks a primary constriction and during anaphase chromatids move as linear bars parallel to the spindle due to almost chromosome-wide spindle attachment (holokinetic behavior; Figure 1B). If a chromosome is monocentric, it bears a primary constriction and the chromatids either move as a v-shaped structure (metacentric), as a linear bar perpendicular to the spindle pole (acrocentric) or as a configuration in between both extreme cases due to microtubule attachment to the defined size-restricted centromere (Figure 1A).

A more direct approach to classify the chromosome structure is through immunolocalization of centromere components. Although many components are functionally conserved, they are often divergent in sequence composition even between closely-related species. Thus, centromere-related antibodies are not universally available. The discovery of phosphorylation of threonine 120 of histone H2A (H2AThr120ph) as universal mark for active centromeres in plant species with mono- and holocentromeres will allow the classification of (comparatively large) chromosomes to be refined (Demidov et al., 2014).

## Holocentromeres in Plants

In flowering plants, holocentromeres are found among the monocots *Cyperaceae* (sedges), *Juncaceae* (rushes; Malheiros et al., 1947; Hakansson, 1958) and *Chionographis* (string flowers; Tanaka, 1977) as well as in dicots such as *Cuscuta* subgenus *Cuscuta* (dodders; Pazy and Plitmann, 1995), *Drosera* (sundews; Sheikh et al., 1995), or in the nutmeg tree *Myristica fragrans* (Flach, 1966). There is evidence for 228 plant species with holocentromeres (Melters et al., 2012). This number is likely an underestimate since for instance many species possess comparatively small chromosomes and thus chromosome classification is cytologically challenging. In addition, all *Juncaceae* and *Cyperaceae* are predicted to be holocentric (the genus *Carex* within *Cyperaceae* consists of around 2000 species, Reznicek, 1990), which actually suggests an even higher number of species with holocentromeres within only these two families. However, there are contradictory reports such as in the genus *Drosera*, where species are reported to possess holocentromeres (Kondo and Lavarack, 1984; Sheikh and Kondo, 1995; Sheikh et al., 1995) and monocentromeres (Shirakawa et al., 2011; Demidov et al., 2014).

## Karyotype Evolution in Species with Holocentromeres

A chromosome-wide centromere organization allows, in theory, rapid karyotype evolution. Unlike monocentric chromosomes, basically each part of a given holocentric chromosome has centromere activity and thus theoretically a high probability of being transmitted during nuclear divisions after chromosome breakage (Figures 1C,D). This karyotype flexibility conferred by a holocentromere is reflected in (i) the extremely wide and almost continuous chromosome number found among related holocentric species, e.g., *Carex* 2n = 12–124, *Eleocharis* 2n = 6–196, or *Juncus* 2n = 18–170 (Bureš et al., 2013), (ii) interspecies chromosome number variation, e.g., *Eleocharis kamtschatica* with 2n = 41–47 (Yano and Hoshino, 2006), or (iii) the negative correlation between chromosome number and chromosome size in *Luzula* (e.g., Nordenskiold, 1951; Jankowska et al., 2015).

Typically double strand breaks (DSBs) in DNA are resolved by non-homologous end-joining or homologous recombination (Knoll et al., 2014). However, telomerase can add telomeric repeats at break sites leading to “chromosome healing” (Tsujimoto, 1993; Tsujimoto et al., 1999; Nelson et al., 2011). Monocentric chromosome healing at DSB sites results in deletion or loss of the distal acentric chromosome fragment and thus needs to be tightly regulated. Unlike monocentric chromosomes, stable transmission of (artificially induced) holocentric chromosome fragments during mitosis and meiosis suggests that chromosome healing is a more or less common phenomenon in holocentric chromosome species (e.g., Hakansson, 1954; Nordenskiold, 1964). Recently, Jankowska et al. (2015) showed that irradiation of holocentric *Luzula elegans* chromosomes results in a range of heteromorphic derived karyotypes. Independent of their size all chromosomes/fragments showed centromere activity and gradual telomere-mediated “chromosome healing.” Newly formed telomere repeats were cytologically detectable 21 days after irradiation in ~50% of cases, increasing to >95% after 3 months. In the progenies of the irradiated plants all the chromosomes/fragments possessed telomeric repeats. This rapid and efficient *de novo* telomere formation is likely conferred by a telomerase-mediated healing process and important for fragment stabilization/karyotype fixation.

The combination of holokinetic chromosomes and rapid telomere formation at DSBs allows stable transmission of chromosome fragments and thus rapid karyotype evolution. Additionally, polyploidy and proliferation/removal of high copy sequences are involved in rapid genome evolution (e.g., Kuta et al., 2004; Zedek et al., 2010; Bozek et al., 2012). However, how holocentric chromosome species can deal with this cytological “chromosomal chaos” is largely unknown. In holocentric *Lepidoptera* with a high intraspecific cytogenetic variation, a high degree of synteny at fine scales is found, suggesting an adaptive mechanism (d’Alencon et al., 2010). Similar studies in plants are missing.

## Structure and Behavior of Holocentric Chromosomes

In plant holocentromeres, cenH3 is found along mitotic chromosomes representing active centromeres as in species with monocentromeres (Nagaki et al., 2005). In some *Luzula* and *Rhynchospora* species with comparatively large chromosomes cenH3-positive chromosome regions form a groove-like structure except at chromosome ends during mitotic metaphase (Nagaki et al., 2005; Heckmann et al., 2011; Cabral et al., 2014; Wanner et al., 2015). It seems likely that a centromeric groove is a structural adaptation of relatively large holocentric chromosomes or a distinct evolutionary accommodation within certain genera. Ultrastructural analysis of mitotic *L. elegans* chromosomes showed that cenH3 containing chromatin is found at the periphery of each individual chromatid and that microtubules attach to cenH3- and not H2AThr120ph-chromatin during mitosis (Wanner et al., 2015). H2AThr120ph is enriched in the centromeric groove and completely absent along the axis where chromatids are in close contact, suggesting that H2AThr120ph is not involved in holocentric sister chromatid cohesion (Wanner et al., 2015). No differentiation between holocentric chromatids is found microscopically in *L. elegans* probably owing to almost chromosome-wide centromeric cohesion (Heckmann et al., 2011; Wanner et al., 2015). Mitotic sister chromatids are only discernible after staining sister-chromatid exchanges (Heckmann and Houben, 2013). Microtubule attachment regions are concentrated on the pericentromeric rims, possibly increasing attachment stability during separation of sister chromatids. Bundles of 2–4 individual microtubules are distributed along the entire centromere length with a mean distance between individual bundles of 300–400 nm during mitosis and 350–500 nm during meiosis (Heckmann et al., 2014a; Wanner et al., 2015). In *C. elegans* with much smaller holocentromeres the number of microtubules attachments is ~85 genome-wide or ~15 per chromosome (O’Toole et al., 2003). In summary, H2AThr120ph and cenH3 are found within the longitudinal centromeric groove of (large) holocentric plant metaphase chromosomes and microtubule attachment is enriched along the groove rim.

The almost chromosome-wide holocentromere architecture is also reflected in the distribution of epigenetic marks. The cell cycle-dependent phosphorylation of serine 10 or serine 28 of H3 typically enriched in pericentromere regions of monocentric plant chromosomes (Houben et al., 1999; Gernand et al., 2003) occurs in *Luzula* and *Rhynchospora* uniformly along the chromosomes (Gernand et al., 2003; Nagaki et al., 2005; Guerra et al., 2006). Similarly typical eu- and heterochromatin epigenetic marks or early/late DNA replicating chromatin domains are detected uniformly along *L. elegans* mitotic chromosomes at normal resolution (Heckmann et al., 2013). However, using super-high-resolution light microscopy interspersed units of various chromatin types were distinguished. Intermingling of different chromatin domains throughout the *L. elegans* genome is correlated with the distribution of highly repetitive DNA and likely reflects interplay between scattered chromosome-wide centromere organization and overall genome organization (Heckmann et al., 2013).

A scattered polycentric centromere arrangement is microscopically reflected in cenH3 dynamics during the cell cycle. During interphase cenH3 is found dispersed, in prophase as small foci along chromosomes and during metaphase as composite linear axial line along each sister chromatid (Buchwitz et al., 1999; Nagaki et al., 2005; Heckmann et al., 2011). In *C. elegans* a polycentric chromosome arrangement is revealed at fine scale resolution (Gassmann et al., 2012; Steiner and Henikoff, 2014). About 700 individual centromeric sites -single cenH3 nucleosomes flanked by well-spaced canonical nucleosomes- are preferentially found at dispersed sites of permissive chromatin (Steiner and Henikoff, 2015). CenH3 is also found with low density in roughly 2900 broad chromosome domains of low transcriptional activity and low nucleosome turnover that put together represent roughly half the genome (Gassmann et al., 2012; Steiner and Henikoff, 2014). Thus, *C. elegans* holocentromeres are polycentromeres consisting of individual point centromeres as the basic units of assembly.

A holo-/polycentric chromosome, composed of various centromeric subdomains, should have a high risk of misorientation during anaphase due to potential merotelic spindle attachment to individual subdomains. In *C. elegans*, chromokinesin KLP-19 counteracts persistent merotelic attachments (Powers et al., 2004). However, it is unclear how holocentric plants circumvent this holocentromere-associated challenge. In dicentric chromosomes, when a critical distance between two active centromeres is reached, the chromosome can break during anaphase due to not forming one functional centromere at metaphase (see below). In case of a holocentric chromosome, the distance between individual centromere subunits must be likewise restricted. In *C. elegans*, with comparatively small holocentric chromosomes, the genomic cenH3 distribution indicates a distance between individual centromere subunits of maximally 1.9 Mb ranging from 290 bp to 1.9 Mb with a median of 83 kb (Steiner and Henikoff, 2014). Notably, during divisions probably not all centromeric regions (only ~15%) are kinetically active in *C. elegans*, thus the maximum distance might be even higher. Similar studies in plants with (larger) holocentric chromosomes are, to date, lacking. Thus, it is unclear what the maximum functional inter-subunit distance tolerated between individual centromere units of a given holocentric plant chromosome is and additionally, it is unclear whether as in *C. elegans*, only a subset of these centromeric domains are kinetically active.

## Holocentromere Identity

Recently, holocentromere-enriched satellite DNA sequences and retrotransposons preferentially bound by cenH3 were found in *R. pubera* (Marques et al., 2015) similar to most plant monocentromeres (Houben and Schubert, 2003; Neumann et al., 2011). Thus, also in species with holocentric chromosomes centromere-specific repetitive DNAs can occur. Stretched *Pisum* chromosomes show multiple centromeres consisting of satellite DNAs (Neumann et al., 2012, 2015). This “meta-polycentricity” may be an evolutionary link toward the development of holocentromeres in species such as *Rhynchospora*.

Unlike *Rhynchospora*, in *L. elegans* neither typical centromere-associated retrotransposons nor any holocentromere-associated satellite DNAs are found (Heckmann et al., 2013). Thus, cenH3 may be associated with a centromere-specific chromatin status rather than with specific centromeric DNA sequences. In *L. nivea* the 178-bp tandem repeat sequence LCS1 (Haizel et al., 2005) which shares some similarity with the centromeric tandem repeat RCS2 of rice (Dong et al., 1998; Nonomura and Kurata, 2001) has been described. Whether LCS1 plays a centromeric role is uncertain.

In *C. elegans* a short DNA motif is enriched at individual centromeric sites, however, it is likely not a direct target for cenH3 (Steiner and Henikoff, 2014). Accordingly, basically any DNA sequence can acquire centromere activity and extrachromosomal arrays are even segregated after few cycles in *C. elegans* (Stinchcomb et al., 1985; Yuen et al., 2011). Thus, in *C. elegans* centromeric nucleosomes are inherited epigenetically rather than being DNA sequence-dependent.

Limited available data suggest that there are different ways of defining holocentromeres with regards to centromeric sequences. In *R. pubera* there are centromere-specific satellite DNAs and retrotransposons, whereas in *L. elegans* and *C. elegans* no centromere-specific sequences are found. Thus, possibly different evolutionary scenarios with regards to centromere-specific DNA sequences led to the formation of holocentromeres. Further studies will clarify, e.g., how in *L. elegans* individual centromeric subunits are defined or whether between closely-related species with holo- and/or monocentromeres such as *Cuscuta* or *Drosera* different or similar centromeric DNA sequences occur.

## Meiotic Adaptations of Holocentric Chromosomes

Sexual reproduction is characterized by the process of meiosis, during which two consecutive rounds of chromosome segregation follow one single round of DNA replication generating haploid gametes. Cytologically, during the first meiotic division homologous chromosomes (homologs) are separated and during the second meiotic division chromatids disjoin. To allow faithful transmission typically homologs pair and perform reciprocal genetic exchange, termed crossover, physically linking homologs and thus ensuring balanced chromosome segregation during meiosis I. Proper chromosome segregation further depends on mono-orientation of fused sister kinetochores during meiosis I and on bi-orientation of sister kinetochores during meiosis II. In monocentromere species this is realized by a two-step loss of cohesion, i.e., along chromosome arms during meiosis I and at sister centromeres during meiosis II.

The two-step loss of cohesion is hampered in a holocentromere due to the lack of defined broad chromosomal centromere and arm domains allowing their spatial distinction. As adaptation, species with holocentromeres evolved different strategies to conduct faithful meiotic chromosome segregation, such as “chromosome remodeling” in *C. elegans* (Schvarzstein et al., 2010) or “functional monocentricity” in *Heteroptera* (Viera et al., 2009). Another alternative meiotic process is found in plants with holocentromeres characterized by separation of sister chromatids already during meiosis I (Cabral et al., 2014; Heckmann et al., 2014a). *Luzula* and *Rhynchospora* display a functional holocentromere throughout meiosis. Prophase I events are cytologically similar to those found in species with monocentric chromosomes including meiotic DSB induction and progression even in achiasmatic chromosomes of *R. tenuis*. At metaphase I, contrary to a monopolar orientation of sister monocentromeres, sister holocentromeres are unfused and interact, individually bi-orientated, with the meiotic spindle, resulting in a separation of sister chromatids already during meiosis I. The homologous non-sister chromatids are kept usually terminally linked by chromatin threads until anaphase II when they are separated ensuring haploidization. These chromatin threads are heterochromatic, enriched in satellite DNAs in *L. elegans* and possibly formed in a crossover-independent manner as their occurrence in achiasmatic chromosomes of *R. tenuis* suggests. Notably, monocentric chromosomes can also associate via chromatin threads (e.g., crane flies or Drosophila, LaFountain et al., 2002; Hughes et al., 2009), suggesting that terminal heterochromatin can be “sticky” enabling an achiasmatic association of homologs and the link of homologous non-sister chromatids in *Luzula* or *Rhynchospora* species. Whether an achiasmatic cohesin-mediated or another unknown mechanism is involved is unclear. Alternatively, these chromatin threads may be formed in a crossover-dependent manner in such a way that only distinct terminal chiasmata persist, being released later than those in interstitial regions. Although *R. tenuis* chromosomes are apparently achiasmatic, DSB and synaptonemal complex formation -prerequisites for crossover formation- suggest that distinct terminal crossovers mediating chromatin thread formation might occur in *R. tenuis*. Additionally, these threads are most likely not caused by catenated late replicating DNA, as homologs and non-sister chromatids are connected as, e.g., in *L. elegans*. Chromatin threads are not described for species with holocentromeres using a different meiotic mode. Therefore, chromatin threads are likely conducive for an inverted meiotic chromatid segregation process in species with holocentromeres (Heckmann et al., 2014b). Interestingly, reminiscent of the situation in monocentric species where centromeres are typically recombination cold spots (Yelina et al., 2012), in species with holocentromeres crossovers mainly occur in non-centromeric (i.e., terminal) chromosome regions. Thus, centromeric regions are crossover cold spots in both mono- and holocentromere species.

## Evolution of Holocentromeres

An intriguing question is whether a holocentric or a monocentric chromosome structure appeared first during evolution. Nagaki et al. (2005) proposed for *Luzula* that a 90°direction turn of a monocentromere in an ancestral *Luzula* species together with subsequent centromere extension could be the basis of holocentricity. Neumann et al. (2012) proposed that spreading of centromere-competent satellite(s) was the cause of a transition from a monocentric to a polycentric chromosome structure in *L. nivea*. Villasante et al. (2007) proposed the “telomere to centromere” model that predicts an origin of holocentromeres from monocentromeres. The “centromere drive” hypothesis suggests a transition from mono- to holocentric chromosomes in order to suppress centromere drive (Malik and Henikoff, 2002). Melters et al. (2012) proposed that monocentricity was the ancestral chromosome configuration and that holocentricity evolved multiple times independently. Currently it is generally accepted that an independent transition from mono- into holocentromeres occurred in total on at least 13 occasions in eukaryotic lineages with the exception of vertebrates (four times in plants and nine times in animals; Melters et al., 2012).

In four holocentric insect lineages cenH3 and CENP-C were independently lost while other outer and inner kinetochore components such as NDC80 or MIS12 remain (Drinnenberg et al., 2014). In *Luzula*, *Rhynchospora*, and *C. elegans* this transition occurred without a loss of cenH3 (Buchwitz et al., 1999; Nagaki et al., 2005; Cabral et al., 2014). Two cenH3s are even found in *C. elegans*, Hcp-3 and Cpar-1 (Monen et al., 2005), and in *L. nivea*, LnCENH3-A and LnCENH3-B (Moraes et al., 2011). CenH3 is essential for mitosis but dispensable for male meiosis in *C. elegans* (Monen et al., 2005). Interestingly, the weakly expressed isoform, Cpar-1, specifically localizes to meiotic chromosomes and is cleaved in its N-terminal tail by separase at anaphase I (Monen et al., 2015). Whether these dynamics reflect a meiotic adaptation compensating for a holocentric chromosome structure during meiosis is unknown. In *L. nivea* two cenH3s are transcribed and at least LnCENH3-B is found in centromeric nucleosomes (Moraes et al., 2011). Whether both cenH3s are essential for centromere activity, are functionally diverged or redundant, and whether this duplication is related to holocentromere occurrence, is at present unclear.

Apparently, a transition from mono- into holocentromeres can evolve differently with regards to cenH3: either entire cenH3 loss in insects, “partial functional loss” being dispensable for male meiosis in *C. elegans*, or complete mitotic and meiotic retention of cenH3 in holocentric plants. Drinnenberg et al. (2014) proposed that an event early in the evolution of insects, e.g., a lineage-specific evolution of a centromeric protein similar to *Umbrea* in flies (Ross et al., 2013), enabled a cenH3 loss in holocentric insects. Unlike holocentric insects, in *L. nivea* and *C. elegans* holocentromeres coincide with two cenH3 variants. Also highly dynamic centromere architectures found in some *Fabeae* species positively correlate with the presence of two active cenH3 variants (Neumann et al., 2015). Possibly two cenH3 variants, albeit also found in monocentric species (e.g., Sanei et al., 2011), either enable or are the consequence of structural centromere changes. Thus, it is tempting to speculate that either any variation in the level of cenH3, loss or duplication, might enable centromere plasticity and thus structural centromere evolution or that structural changes in centromere architecture may render centromere-dependency on cenH3. In a nutshell, transitions from mono- into holocentromeres are likely based on distinct evolutionary scenarios rather than on one common pivotal event.

In *Cuscuta*, plants of the subgenera *Monogyna* and *Grammica* are reported to have monocentromeres while members of the subgenus *Cuscuta* are reported to have holocentromeres (Pazy and Plitmann, 1994, 1995). Similarly, some *Drosera* species have monocentromeres (Shirakawa et al., 2011; Demidov et al., 2014) while others are reported to have holocentromeres (Kondo and Lavarack, 1984; Sheikh and Kondo, 1995; Sheikh et al., 1995). These species potentially offer a great opportunity to gain further insight into the evolution of differing centromere types between closely-related species and of the mechanisms involved. Additionally, occurrence of holo- and monocentromeres between closely related *Cuscuta* or *Drosera* species might offer a possibility to generate hybrids between species with holo- and with monocentromeres. This would be an attractive model system to study two different centromere types within a hybrid.

Another striking question is why holocentricity has arisen multiple times during evolution in diverse eukaryotic lineages but not in all? One common explanation is their advantage in relation to DSBs when compared to monocentric chromosomes. However, widespread occurrence of monocentromeres and the fact that holocentromeres are not described (so far) in vertebrates suggest that potential advantages conferred by a holocentromere are counteracted by certain disadvantages; possibly the potential of merotelic chromosome attachments during nuclear divisions or the faithful segregation of meiotic chromosomes. This may explain why species with holocentromeres evolved diverse meiotic chromosome segregation processes and why manifold mechanisms for sexual and/or asexual reproduction are found in species with holocentromeres (e.g., in aphids, Manicardi et al., 2015).

## Di-, tri- and meta-polycentric Chromosomes

**FIGURE 2:**
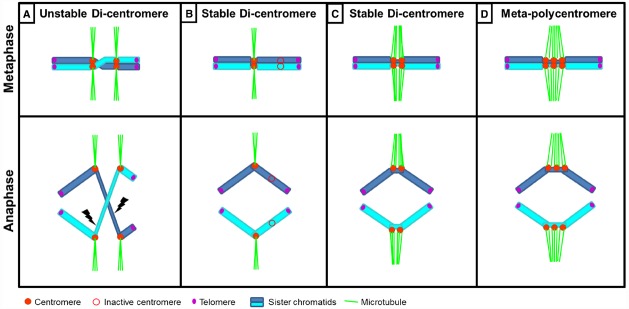
**Schematic representation of chromosomes with multiple centromeres. (A)** A chromosome with two active centromeres (di-centromere or dicentric chromosome) is typically unstable. A twist between sister chromatids within the region between both centromeres leads to merotelic spindle attachment to two kinetochores on the same chromatid resulting in an anaphase bridge and subsequent chromosome breakage. **(B,C)** Stabilization of a dicentric chromosome can occur by **(B)** inactivation of one centromere, so that the chromosome behaves as monocentric or **(C)** when the close proximity between two active centromeres enables them to behave as one functional unit. **(D)** Meta-polycentric chromosome with three functional centromeres within one constriction.

Dicentric chromosomes (chromosomes with two active centromeres) are typically unstable (Figure 2A). They form anaphase bridges which lead to chromosome breakage, as already observed by Barbara McClintock (1939) in maize. Dicentric chromosomes are reported in plant and non-plant species such as *Drosophila*, yeast and human (Stimpson et al., 2012). There are even reports about tricentric (with three centromeres; Zhang et al., 2010) and meta-polycentric (with up to five centromeres) chromosomes (Neumann et al., 2012, 2015). Typically, di- or tricentric chromosomes arise as a consequence of profound genome rearrangements (Stimpson et al., 2012) although naturally occurring di- and meta-polycentric chromosomes do exist.

Stabilization of di- and tricentric chromosomes can occur via different mechanisms. One mechanism is the epigenetic inactivation of one of the centromeres leading to a functional monocentric chromosome (Figure 2B). If two or three centromeres are different in size, the small centromere(s) is/are inactivated (Han et al., 2009a; Zhang et al., 2010). An inactivated centromere can be reactivated when detached from the active centromere, demonstrating that it retains centromeric capability (Schubert et al., 1995; Han et al., 2009a). Stabilization can also occur if one centromere exhibits functional dominance over the other(s). A tricentric wheat chromosome (Zhang et al., 2010) was found with one large and two small centromeres that due to being in close proximity, function as one unit. All three centromeres contain centromeric sequences, cenH3 (the small centromeres containing 30% the amount of the large centromere), H3S10ph and bind spindle microtubules. This tricentric chromosome exhibits features of dicentrics: the two smaller centromeres can be inactivated, positively correlating with increased amounts of H3K27me2/3 or, when the two smaller centromeres are active, chromosome breakage occurs. However, in 70% of progenies the intact tricentric chromosomes was transmitted, possibly due to dominant pulling forces of the large over the small centromeres during anaphase (Zhang et al., 2010).

Stabilization is further dependent on proximity between the two centromeres: if they are “close enough,” both active centromeres can behave as a functional unit and orientate to the same pole (Figure 2C), whereas when a critical distance is reached between the two active centromeres the chromosome can break due to merotelic spindle attachments. The critical distance is estimated to be around 10 Mb in a human X chromosome (Sullivan and Willard, 1998) and up to 20 Mb in an engineered human dicentric chromosome (Higgins et al., 2005). A naturally occurring stable “dicentric” chromosome is found in rice (Wang et al., 2013). Two cenH3-binding domains composed of typical centromeric repeats are separated by ~400 kb 5S rDNA sequences that do not associate with cenH3. Also canonical centromeres contain blocks of H3- instead of cenH3-containing chromatin of considerable size, e.g., rice Cen8 (Nagaki et al., 2004; Yan et al., 2008), potato Cen9 and Cen11 (Gong et al., 2012) or “dicentric” maize chromosome 5 with an estimated “gap” of 2.8 Mb (Wolfgruber et al., 2009). In the most extreme case, meta-polycentric chromosomes in pea and closely related *Lathyrus* species contain three to five functional centromeres within stretched primary constrictions (Neumann et al., 2012, 2015; Figure 2D). In these meta-polycentric chromosomes of pea up to several megabases are estimated to lie between cenH3-containing domains (Neumann et al., 2012). Thus, it seems likely that as long as the distance between multiple centromeres per chromosome is limited, these centromeres can function together, similar to the situation found in holocentric chromosomes.

## Neocentromeres

**FIGURE 3:**
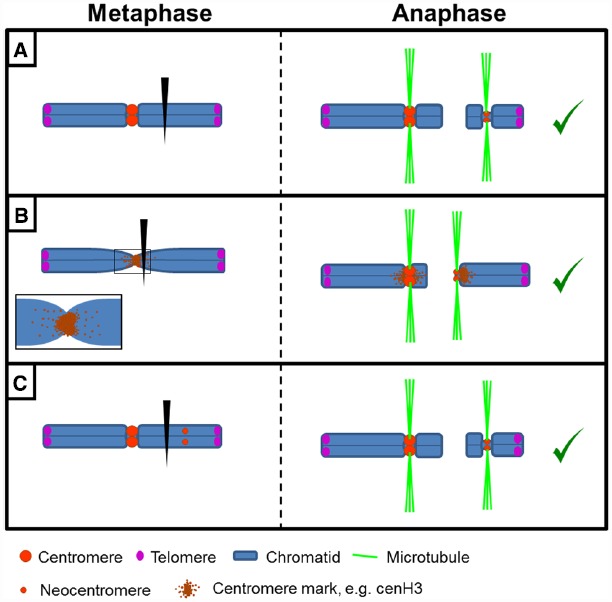
**Formation and behavior of ***de novo*** centromeres. (A)** Following chromosome breakage, an acentric fragment can form a neocentromere allowing its proper transmission. **(B)** Chromosome breakage close to the endogenous centromere may lead to neocentromere formation due to presence or spreading of centromeric marks (e.g., cenH3) to pericentromeric regions. **(C)** Neocentromere formation in an intact chromosome leads to a dicentric chromosome structure. If this results in chromosome breakage, a centromere- and a neocentromere-containing fragment will result.

**FIGURE 4:**
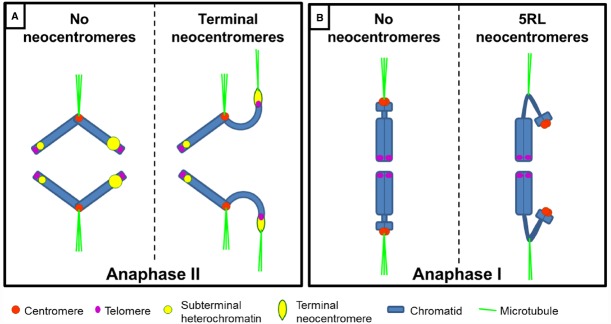
**Schematic representation of meiosis-specific neocentromeres in plants. (A)** During anaphase II, terminal neocentromeres are visible as heterochromatic stretches directed toward the cell poles ahead of the centromere. Telomeric regions are not stretched to the poles. Large heterochromatic regions (represented as larger yellow circles) are more prone to form neocentromeres than small ones. **(B)** Neocentromere in rye 5RL arises at an interstitial heterochromatic constriction and can substitute for the centromere during anaphase I.

A neocentromere is a chromosomal locus outside the endogenous centromere that acquires kinetic activity. They are described in various organisms including plants, yeast, flies, chicken and humans (Guerra et al., 2010; Burrack and Berman, 2012; Scott and Sullivan, 2014). The term “neocentromere” has been traditionally used to define two different phenomena: (i) a *de novo* centromere formation occurring after chromosome breakage or endogenous centromere inactivation, which allows transmission of the (re-arranged) chromosome (Figure 3), and (ii) the kinetic motility of terminal or subterminal heterochromatin, which is pulled to the cell poles during meiosis in plants (Figure 4).

## *De novo* Centromeres

In monocentric chromosome species occasionally an acentric chromosome fragment is stably transmitted during mitosis and meiosis due to neocentromere formation (Figures 3A,B).

In non-plant species, human neocentromeres are the best described to date. They usually appear in rearranged chromosomes associated with developmental delays or cancer and are typically isolated from clinical samples (Burrack and Berman, 2012). The first human neocentromere was reported by Voullaire et al. (1993) in chromosome 10. To date, neocentromeres are found in all human chromosomes except chromosome 19 (Marshall et al., 2008; Liehr et al., 2010; Klein et al., 2012), typically in euchromatic regions devoid of alpha-satellite DNAs characteristic of human centromeres (Alonso et al., 2010). These initial findings suggested that (alpha) satellite DNAs and/or heterochromatin are not necessary for (human) centromere activity. However, like endogenous centromeres, human neocentromeres form a primary constriction and contain all tested centromere-associated proteins except CENP-B whose localization is sequence-specific and requires a CENP-B box found in endogenous centromeres (Saffery et al., 2000).

In plants, *de novo* centromere formation has been documented in barley and maize. They appear in rearranged acentric chromosomes devoid of typical centromeric DNA sequences but contain centromeric proteins including cenH3 (Nasuda et al., 2005; Topp et al., 2009; Fu et al., 2013; Zhang et al., 2013; Liu et al., 2015).

In a barley telocentric chromosome derived from a 7HS isochromosome a neocentromere occurred close to the endogenous centromere (Nasuda et al., 2005). In maize, two *de novo* centromeres were reported in chromosome 3, one in the short arm (3S, Topp et al., 2009) and another one in a derivative of the long arm called Dp3a (Fu et al., 2013). In maize 3S cenH3 amounts varied between lines and low amounts of cenH3 positively correlated with low transmission rates. CenH3 levels increased over generations and the neocentromere became more stable, thus accumulation of cenH3 over time likely stabilizes the neocentromere (Topp et al., 2009). The neocentromere in Dp3a formed within protein-coding genes (Fu et al., 2013), similar to, e.g., rice centromere 8 which contains actively transcribed genes (Nagaki et al., 2004; Yan et al., 2008). Although Dp3a was occasionally transmitted during meiosis, it got frequently lost during somatic divisions suggesting that the neocentromere was unstable. The cenH3-binding region was ~350 kb, considerable smaller than the megabase-sized cenH3 binding domains of canonical maize centromeres (Wolfgruber et al., 2009). Possibly the amount of cenH3 within the 350 kb is not sufficient for proper centromere activity and larger cenH3 amounts, potentially acquired over successive generations (Topp et al., 2009), are required for stabilization of neocentromeres. Neocentromeres are also found in supernumerary maize B chromosomes (Zhang et al., 2013; Liu et al., 2015) indicating that they are not exclusive to A chromosomes.

## Origin of *de novo* Centromeres

Why these neocentromeres appear and why specifically in a given chromosome region is an intriguing question. *De novo* neocentromeres might represent “latent” centromeres (Voullaire et al., 1993; Choo, 1997), locations of ancestral centromeres following centromere repositioning events (Rocchi et al., 2012). Alternatively, spreading of centromeric chromatin “marks” to adjacent chromosomal regions may explain neocentromeres arising close to an endogenous centromere (Maggert and Karpen, 2001; Nasuda et al., 2005; Shang et al., 2013; Thakur and Sanyal, 2013; Zhang et al., 2013; Figure 3B). In chicken, for instance, cenH3 is found in pericentromeric regions and that could trigger neocentromere formation when the endogenous centromere is removed (Shang et al., 2013).

For neocentromeres located far away from the canonical centromere, an interesting point arises: are they a *consequence*, or conversely a *cause*, of chromosome breakage? Generally a neocentromere is proposed to “rescue” acentric chromosome fragments allowing their transmission during cell divisions (hence, they would be a *consequence*). However, neocentromere formation could also be the *cause*. If a new region acquires the ability to assemble an active kinetochore, this could lead to a dicentric structure and subsequent chromosome breakage (Figure 3C). Possibly, both options exist: whereas experimentally centromere excision results in neocentromere formation (Ishii et al., 2008; Ketel et al., 2009; Shang et al., 2013; Thakur and Sanyal, 2013), artificially tethering of centromeric components to non-centromeric loci or overexpression of cenH3 can lead to ectopic kinetochore formation and chromosome instability (Heun et al., 2006; Barnhart et al., 2011; Mendiburo et al., 2011; Olszak et al., 2011; Teo et al., 2013). CenH3 is also found in non-centromeric chromatin in, e.g., human and chicken cells (Shang et al., 2013; Bodor et al., 2014) but whether these ectopic cenH3 domains can nucleate under certain conditions a *de novo* centromere is unclear. It has been proposed that small neocentromeres could be nucleated with relatively high frequency. While presence of the endogenous larger centromere prevents them from becoming active, in fragments detached from the main centromere this activation could occur (Liu et al., 2015).

## Terminal Neocentromeres in Plants

Terminal neocentromeres were first described by Kattermann (1939) in rye who called them “T-chromosomes” for “terminal chromosomes.” He described an activity in chromosomes from inbred lines resembling terminal centromeres. Prakken and Müntzing (1942) and Rhoades and Vilkomerson (1942) described in rye and maize respectively, that chromosome ends were *attracted* to the poles. The term “neo-centric” was used for the first time by Rhoades (1942). Later reports described similar chromosome activities, primarily in grasses but also in lilies and a moss (Dawe and Hiatt, 2004; Figure 4A).

Maize terminal neocentromeres appear in heterochromatic DNA domains known as knobs (Rhoades and Vilkomerson, 1942). Under standard conditions, knobs are inert. However, presence of a chromosome 10 carrying a distinct large knob called abnormal 10 (Ab10) renders them active as neocentromeres during meiosis (Rhoades and Vilkomerson, 1942). Ab10-dependent terminal neocentromere activity causes the preferential transmission - meiotic drive- of knobbed chromosomes to the egg cell during female meiosis (Rhoades, 1942). Terminal neocentromeres are genetically regulated by a locus called *Smd1* (suppressor of meiotic drive 1; Dawe and Cande, 1996) and further unknown genes (Hiatt et al., 2002; Hiatt and Dawe, 2003). Smd1 regulates the activation of terminal neocentromeres and their subsequent preferential segregation (Dawe and Cande, 1996). Interestingly, terminal neocentromeres are necessary but not sufficient for the preferential transmission of Ab10 and knobbed chromosomes since deletion of *smd1* led to neocentromere activity without meiotic drive (Dawe and Cande, 1996). Moreover, the two major components of maize knobs, i.e., a 180-bp satellite repeat and a 350-bp tandemly repeated sequence (TR-1; Peacock et al., 1981; Ananiev et al., 1998; Gonzalez-Sanchez et al., 2007) are independently regulated in such a way that both repeats “compete” genetically to be preferentially transmitted (Hiatt et al., 2002; Kanizay et al., 2013). Thus, maize neocentromeres might constitute a mechanism by which knobs and associated loci are preferentially transmitted in a process resembling the behavior of “selfish” B chromosomes (Houben et al., 2014).

Maize terminal neocentromeres lack, based on immunological analysis, cenH3 and CENP-C (Dawe et al., 1999; Dawe and Hiatt, 2004) but interact with spindle microtubules albeit in a lateral way and not the canonical end-on fashion (Yu et al., 1997). The poleward motility of chromosome arms is not affected by the microtubule-stabilizing drug taxol suggesting that a meiosis specific microtubule-based motor protein could be responsible for their kinetic activity (Hiatt et al., 2002). Identification of such protein remains elusive.

Rye terminal neocentromeres were initially observed in inbred populations (Kattermann, 1939; Prakken and Müntzing, 1942) and later also in open pollinated rye varieties (Kavander and Viinikka, 1987; Manzanero and Puertas, 2003). During meiosis, all rye chromosomes can show terminal neocentromeres that interact with microtubules (Puertas et al., 2005) but they preferentially occur in chromosomes with large C-banding positive heterochromatic blocks (Manzanero and Puertas, 2003; Figure 4A). The repetitive sequences pSc200 and pSc250 (Vershinin et al., 1995), but not centromeric or telomeric sequences, are stretched at the neocentromeres (Manzanero and Puertas, 2003). Terminal heterochromatin domains of rye B chromosomes show no neocentromeric activity (Manzanero and Puertas, 2003). Thus, only a subset of terminal heterochromatin can acquire kinetic activity. Similar to maize, rye terminal neocentromeres are genetically regulated. Hayward (1962) reported that rye neocentromeres are controlled by a polygenic system and Puertas et al. (2005) proposed a model based on two *trans*-acting genes. Contrary to maize, no examples of meiotic drive are reported for rye terminal neocentromeres.

Terminal neocentromeres are active together with the endogenous centromere stretching the chromosome arms to the same, or the opposite pole of the centromere (Manzanero and Puertas, 2003). Thus, chromosomes behave as di- or polycentric during meiotic divisions but chromosome breakage does not occur and the endogenous kinetochore leads the chromosome movement to the poles (Yu et al., 1997; Manzanero and Puertas, 2003).

Initial observations in maize suggested that terminal neocentromeres could enable migration of acentric fragments to the cell poles (Rhoades and Dempsey, 1966; Dawe and Cande, 1996). However, it was later demonstrated that only when an acentric fragment was brought to the equator plate (possibly by interaction with other bivalents) could it then migrate to the pole during anaphase I (Yu et al., 1997). Similarly, rye terminal neocentromeres fail to move acentric fragments unless they are physically linked (even by chromatin threads) to a centromere (Puertas et al., 2005). Therefore, a *cis*-acting centromere is essential for terminal neocentromere activity.

## Origin of Terminal Neocentromeres

Why would the chromosome ends acquire kinetic activity during meiosis? Frequently terminal neocentromeres appear in situations of genetic instability, such as inbred populations, interspecific hybrids, heat stress, X-ray irradiated plants or presence of abnormal chromosomes (Dawe and Hiatt, 2004). However, they also appear in normal cultivars of diploid rye. Neocentromeres in fission yeast (Ishii et al., 2008), *Drosophila* (Platero et al., 1999) and human (Marshall et al., 2008) preferentially occur in subtelomeric regions. This could indicate that subtelomeres are preferred domains to acquire centromeric activity or, in line with the “centromeres from telomeres” model (Villasante et al., 2007), that subtelomeric regions might have retained some of the ancestral features which (especially under unusual genomic conditions) allow them to recover centromeric activity. Recently, an interesting functional exchange of roles between centromeres and telomeres during yeast meiosis was found (Fennell et al., 2015): in mutants that fail to form a meiotic bouquet (attachment of telomeres to the nuclear envelope during meiotic prophase, essential for proper meiotic progression in some species) centromeres can functionally replace telomeres and (at least partially) rescue the mutant phenotype. This indicates that centromeres and telomeres are not completely independent functional entities.

Kinetochore-independent chromosome movements similar to terminal neocentromeres are also found in other organisms. For example during mitotic metaphase in the African blood lily *Haemanthus katherinae* a poleward force acts on chromosome arms facilitating movement of acentric chromosome fragments (Khodjakov et al., 1996), or in the germ line of *Parascaris*, large amounts of satellite DNAs (heterochromatin) are enriched at its chromosome termini functionally acting as centromeres but getting eliminated rapidly in somatic tissues (Pimpinelli and Goday, 1989). Whether similar mechanisms are involved in the regulation of terminal neocentromere activity is unclear.

## Other Types of Neocentromeres

Key differences between *de novo* and terminal neocentromeres are that the latter do not mediate sister chromatid cohesion nor lead chromosome movement in the absence of an active endogenous centromere (Table 1). An intermediate situation is found in the long arm of chromosome 5 of rye (5RL; Figure 4B). A neocentromere in 5RL is found that is active only during meiosis, can coincide with the centromere(s) and is associated with heterochromatin (Manzanero et al., 2000, 2002), similar to terminal neocentromeres. However, unlike terminal neocentromeres, it mediates sister chromatid cohesion at anaphase I and leads chromosome movement when the endogenous centromere is inactive, similar to the situation found in *de novo* centromeres (Manzanero et al., 2000, 2002; Cuacos et al., 2011).

**TABLE 1:** **Comparison of the three types of neocentromeres described with regards to their main features**.

**Feature**	***De novo* centromere**	**Terminal neocentromere**	**5RL neocentromere**
Replaces the centromere	Yes	No	Occasionally
Sister-chromatid cohesion	Yes	No	Yes
Visible constriction	Yes	No	Yes
Genomic location	Eu- and heterochromatin	Heterochromatin	Heterochromatin
Centromeric proteins	Yes	No	No
Interaction with spindle microtubules	Yes (end-on)	Yes (lateral in maize, end-on in rye)	Yes (end-on)
Species	Human, chicken, yeast, Drosophila, barley, maize	Plants (e.g., maize, rye)	Rye, wheat-rye addition lines

This neocentromere arises in an interstitial constriction in haploid rye, wheat-rye and wheat-Triticale hybrids and wheat-rye addition lines involving chromosome 5R, both in mono(telo)somic and di(telo)somic conditions (Schlegel, 1987; Manzanero et al., 2000, 2002; Cuacos et al., 2011). When the 5RL neocentromere is active, the constriction is cytologically stretched up to several times the chromosome length, reaching the cell poles before the onset of anaphase I (Manzanero et al., 2000, 2002; Cuacos et al., 2011). Interestingly, it can lead chromosome movement together with the centromere or alone (Manzanero et al., 2000, 2002; Cuacos et al., 2011) suggesting that this neocentromere, unlike terminal neocentromeres, does not require a *cis*-acting centromere to be active (Figure 4B).

The heterochromatic 5RL interstitial constriction lacks typical centromeric and telomeric sequences but contains the repetitive sequences pSc119.2 (Bedbrook et al., 1980; McIntyre et al., 1990) and UCM600 (Manzanero et al., 2002; Gonzalez-Garcia et al., 2011; Cuacos et al., 2011). Proteins accumulate at the constriction and a thin bundle of microtubules is end-on attached (Manzanero et al., 2002; Cuacos et al., 2011), showing that interaction between spindle microtubules and the constriction occurs. Notably, kinetic activity is cenH3- and CENP-C-independent (Cuacos, 2013) similar to maize terminal neocentromeres.

Varying frequencies of active rye 5RL neocentromeres in wheat-rye addition lines (Manzanero et al., 2000, 2002) suggested the influence of an environmental factor in its activity. Indeed, treating plants with an organophosphate pesticide significantly increased a basal frequency of ~10% neocentromeres up to ~50% (Cuacos et al., 2011). Concomitantly, an alteration of the meiotic spindle was found possibly facilitating the interaction of the spindle microtubules with the constriction (Cuacos et al., 2011). However, why this region acquires under certain conditions such ability is not well understood.

In *Bromus marginatus* and *B. pseudolaevipes* hybrids (Walters, 1952), *Aegilops markgrafii* (Schubert, 2011), and wheat chromosomes in the progeny from Triticale × tritordeum hybrids (Carvalho et al., 2008) a stretched constriction during meiosis, similar to the one found in rye 5RL, was described. In *A. markgrafii* this constriction is not related to neocentromeric activity since cenH3 and microtubules are exclusively found at the endogenous centromere (Schubert, 2011). Whether constrictions in *Bromus* or wheat acquire kinetic activity is not documented.

Heterochromatin within the 5RL neocentromere could explain its ability to maintain sister chromatid cohesion, as heterochromatin within pericentromeric regions does in native centromeres (Bernard et al., 2001; Nonaka et al., 2002). Moreover, a heterochromatic environment is likely conducive for neocentromere formation, as fission yeast (Ishii et al., 2008) and *Drosophila* (Platero et al., 1999; Olszak et al., 2011) neocentromeres are commonly found in heterochromatic regions. Also human neocentromeres arising at euchromatic domains recruit heterochromatic proteins (Saffery et al., 2000) and plant terminal neocentromeres are heterochromatin-associated. However, the latter do not maintain sister chromatid cohesion suggesting that heterochromatin is necessary but not sufficient to maintain sister chromatid cohesion.

## Neo-centromere Formation and Evolution

Different models have been proposed to explain the origin of the centromere. Villasante et al. (2007) proposed that centromeres were derived from telomeres, in such a way that subtelomeric regions were the first proto-centromeres recognized by spindle microtubules. This model is, e.g., supported by telomere-like sequences found within centromeric regions of several species (Villasante et al., 2007 and references therein). The model assumes that centromeres were, from their origin, associated with repetitive DNA elements.

Another model proposes the opposite: centromeres originally formed on single-copy non-repetitive DNA loci and subsequently acquired highly repeated sequences (Gong et al., 2012; Rocchi et al., 2012). In support of this model, repeat-free centromeres have been found. For example in rice, the centromere of chromosome 8 contains low amounts of satellite DNAs (Nagaki et al., 2004) and some species lack the centromeric satellite CentO (Lee et al., 2005). Also, in potato five centromeres contain unique- or low-copy repetitive DNA (Gong et al., 2012). In non-plant species, repeat-less centromeres are found in chicken (Shang et al., 2010) and the genus Equus (Wade et al., 2009). This model is further supported by the fact that centromere repositioning has occurred in mammals and plants, i.e., a new centromere formed in a different chromosome region while the “old” one became inactive (Han et al., 2009b; Rocchi et al., 2012). Consistently, shattered remnants of centromeric satellites are found throughout plant and animal genomes (Rocchi et al., 2012; Lysak, 2014).

In potato, together with repeat-free centromeres there are centromeres containing megabases of repetitive satellite DNA with an extremely large monomer size up to 5 kb (Gong et al., 2012) which are not, or only rarely found in closely related species (Gong et al., 2012; Wang et al., 2014b; Zhang et al., 2014) suggesting a relatively recent species-specific amplification. The coexistence of centromeres with and without repetitive DNA suggests that accumulation of repeats could be a sudden, rather than progressive, process. These sequences could be incorporated from another centromere or constitute new repeats, for which retrotransposons might play a relevant role (Gong et al., 2012; Gao et al., 2015). Later accumulation or expansion of repeats could occur by several mechanisms such as replication slippage, rolling circle replication followed by reinsertion or transposition. Moreover, maize centromeric satellites are highly diverged from ancient maize centromere satellites and chromosome-specific satellites are not found, suggesting existence of a homogenization mechanism (Bilinski et al., 2015). Finally, maize chromosomes transferred to a species with larger genome such as oat experience a rapid expansion of centromere size (cenH3-binding domain; Wang et al., 2014a) agreeing with previous observations that correlated centromere and genome size in grasses (Zhang and Dawe, 2012). Altogether, this demonstrates that neo-centromere evolution can be highly dynamic.

## Concluding Remarks

Despite striking progress made in plant centromere biology, many questions remain to be answered. How could such an essential chromosomal locus evolve structurally so diverse, from a point centromere up to a holocentromere? The more data on centromeres becomes available, the more challenging it is to define the centromere. The centromere could be best described as a “conserved function, but distinct structure, organization and features between organisms.” In future, a better understanding in different organisms and in different centromere types of the precise nature of epigenetic mechanisms specifying centromere location, formation and maintenance as well as of the unsolved connection between epigenetics and genetics behind centromere biology is needed.

We anticipate that further studies of (plant) species with atypical centromeres will broaden our mainly monocentric chromosome-biased knowledge on centromere definition and will help in establishing more accurate models of centromere biology and evolution. Future research on plant centromere biology will also help plant genetic engineering (e.g., artificial chromosome or double haploid plant production) and thus ultimately help plant breeding.

### Conflict of Interest Statement

The authors declare that the research was conducted in the absence of any commercial or financial relationships that could be construed as a potential conflict of interest.
